# A Sex Difference in the Predisposition for Physical Competition: Males Play Sports Much More than Females Even in the Contemporary U.S

**DOI:** 10.1371/journal.pone.0049168

**Published:** 2012-11-14

**Authors:** Robert O. Deaner, David C. Geary, David A. Puts, Sandra A. Ham, Judy Kruger, Elizabeth Fles, Bo Winegard, Terry Grandis

**Affiliations:** 1 Department of Psychology, Grand Valley State University, Allendale, Michigan, United States of America; 2 Department of Psychological Sciences, University of Missouri, Columbia, Missouri, United States of America; 3 Interdisciplinary Neuroscience Program, University of Missouri, Columbia, Missouri, United States of America; 4 Department of Anthropology, Pennsylvania State University, University Park, Pennsylvania, United States of America; 5 Sandra Ham Consulting, Chicago, Illinois, United States of America; 6 Department of Environmental Health, Rollins School of Public Health, Emory University, Atlanta, Georgia, United States of America; 7 Department of Psychology, Florida State University, Tallahassee, Florida, United States of America; 8 Department of Psychology, State University of New York at New Paltz, New Paltz, New York, United States of America; Australian Wildlife Conservancy, Australia

## Abstract

Much evidence indicates that men experienced an evolutionary history of physical competition, both one-on-one and in coalitions. We thus hypothesized that, compared to girls and women, boys and men will possess a greater motivational predisposition to be interested in sports, especially team sports. According to most scholars, advocacy groups, and the United States courts, however, this hypothesis is challenged by modest sex differences in organized school sports participation in the contemporary U.S., where females comprise 42% of high school participants and 43% of intercollegiate participants. We conducted three studies to test whether organized school sports participation data underestimate the actual sex difference in sports participation. Study 1 analyzed the American Time Use Survey, which interviewed 112,000 individuals regarding their activities during one day. Females accounted for 51% of exercise (i.e., non-competitive) participations, 24% of total sports participations, and 20% of team sports participations. These sex differences were similar for older and younger age groups. Study 2 was based on systematic observations of sports and exercise at 41 public parks in four states. Females accounted for 37% of exercise participations, 19% of individual sports participations, and 10% of team sports participations. Study 3 involved surveying colleges and universities about intramural sports, which primarily consist of undergraduate participation in team sports. Across 34 institutions, females accounted for 26% of registrations. Nine institutions provided historical data, and these did not indicate that the sex difference is diminishing. Therefore, although efforts to ensure more equitable access to sports in the U.S. (i.e., Title IX) have produced many benefits, patterns of sports participation do not challenge the hypothesis of a large sex difference in interest and participation in physical competition.

## Introduction

A game can be defined as an organized activity where two or more sides compete according to agreed-upon rules, and a sport can be defined as a game that requires physical skill (see [1]–[3]). Sports occur in most or all human societies [1], [3]–[6], and numerous functions have been hypothesized, all of which appear mutually compatible [1], [7]. However, from an evolutionary perspective (i.e., linked to survival and reproduction), three hypotheses seem plausible [7]. First, sports may function as culturally invented courtship rituals that reliably advertise participant quality to the opposite sex [8], [9]. Second, sports may function as physical competitions for status, differing from unrestrained combat or warfare because they reduce the risk of physical harm to competitors and more publicly and efficiently reveal the competitors’ competitive qualities [7], [8], [10]. Third, sports may function to build skills necessary for physically-demanding activities, especially combat, warfare, and hunting [2], [11]–[13]. Although these hypotheses are based on adaptive logic, none require the claim that sports are an adaptation *per se*. Instead they assume (or allow) that sports arise as byproducts of other adaptations, including motives and capacities to physically compete for mates and status, negotiate and enforce behavioral norms, and monitor the abilities of potential competitors, mates and allies.

To the extent that these hypotheses hold, especially the second and third ones, we further hypothesize that, compared to girls and women, boys and men will, on average, have a far greater inborn motivational predisposition to participate in and monitor sports, especially sports involving combat-relevant skills and/or team play. This hypothesis follows from the following points. First, many lines of evidence indicate that throughout human evolutionary history and during contemporary periods, men have been substantially more likely than women to engage in contests involving extreme physical aggression [14]–[17], between-group raiding and warfare [18]–[20], and cooperative hunting [21], [22]. Indeed, many sports require skills relevant for combat or hunting, such as running, tackling, and throwing or dodging projectiles [2], [6], [12]. In addition, some sports involve two teams competing against each other, with team play often requiring the differentiation of roles, coordination among teammates, and tactical planning [2], [6].

Second, a history of male-male aggression is revealed by pronounced sexual dimorphism in musculature, strength [23], [24], and speed [25], [26]. Similarly, men (but not women) possess secondary sexual characteristics (e.g., beards, pronounced jaws, deep voices) that function to threaten rivals [27]–[30]. Another legacy of this history is a predisposition(s) to behaviorally prepare for physical contests, both individually and in groups. This is indicated by the fact that, in all societies that have been studied, boys engage in more rough-and-tumble play and play-fighting [31]–[35]. Boys are also more likely to form large same-sex groups, to differentiate roles within such groups, and to seek competition with other groups [32], [36], [37]. Moreover, several kinds of evidence indicate that these sex-differentiated play patterns are due, at least in part, to boys’ typically greater exposure to androgens prior to birth [38].

Much evidence supports the hypothesis that males are more predisposed to be interested in sports. First, historical reviews of sports document that many societies had substantial female participation, but males are reported as being much more involved in most or all cases [2], [6], [39]. Similarly, cross-cultural ethnographic studies of sports have focused on male sports, apparently due to their greater prominence (e.g., [3], [5], [12], [13]. In fact, a recent study found there were more male sports than female sports in all societies in the Human Relations Area Files probability sample [40] (see also [41]). Second, studies in large contemporary societies ubiquitously report greater male interest in participating, watching, and excelling in sports. Evidence comes from self-reports of interest (summarized in [16]) and from actual participation (e.g., [42]–[44]). Third, several studies have reported that females with congenital adrenal hyperplasia, a disease characterized by heightened prenatal androgen exposure, are more likely than unaffected females to show strong interest in stereotypically masculine sports [45]–[47].

It is important to emphasize that this evolutionary hypothesis is fully compatible with research implicating social influences on sports interest. For example, adolescent females often experience great pressure to eschew sports, especially stereotypically masculine sports [48]–[50]; this can be understood in terms of female mating competition, which generally emphasizes femininity [51], [52]. Conversely, males may be rewarded for embracing masculine norms in sports [53]–[55]; this makes sense given that, for males, excelling in stereotypically masculine sports is associated with greater mating success [2], [7], [56](see also [10], [57], [58]). Although the particular patterns of inculcation can be expected to differ across societies, an evolutionary perspective suggests that socialization will generally amplify inborn predispositions associated with sex [32], [59] (see also [60]).

### The Challenge of Title IX

Despite the evidence for a greater male than female predisposition for sports interest, a powerful challenge to this hypothesis has emerged in the form of surging sports participation by girls and women in some contemporary nations. The most striking example is in the United States, where female participation in organized sports has grown steadily over the past four decades. For instance, in 1972 females comprised 7% of high school athletes, whereas in 2010, they comprised 42% [61]; in NCAA intercollegiate sports, females comprised 30% of athletes in 1982 and 43% in 2009 [62]. These changes occurred after the enactment in 1972 of a federal law, known as Title IX, that prohibits sexual discrimination in educational opportunities, including sports, and that resulted in the creation of substantially more equitable opportunities and incentives (e.g., scholarships) for female athletes [63]–[65].

Most scholars, advocacy groups, and the U.S. courts have interpreted the substantial participation gains as indicating that females’ sports interest is intrinsically equal to that of males and that opportunities following in the wake of Title IX merely allowed females to express their interest. This “If you build it, they will come” logic is the bedrock of current Title IX interpretation and execution [63]–[65]. For example, *The Women’s Sports Foundation*, the United States’ most influential organization in advancing “the lives of girls and women through sports and physical activity” has a section on its website called “Title IX Myths and Facts” and it states:

“MYTH: Girls are not as interested as boys in playing sports. FACT: The dramatic increase in girls’ and women’s participation in sport since Title IX was passed in 1972 (by 545% at the college level and 979% in high schools) demonstrates that it was lack of opportunity – not lack of interest – that kept females out of high school and college athletics for so many years.” [66].

The surge in female sports participation in the U.S. is certainly remarkable. Nevertheless, equal or nearly equal participation in organized school sports does not necessarily equate with equal or nearly equal interest in sports. One reason is that, on average, a participating male, compared to a participating female, may have a stronger interest in or valuation of sports or may have a stronger desire to be a sports spectator. Self-report studies conducted in the U.S. consistently support these suggestions, both for interests [67]–[70] and fandom [71]–[74]. However, counter interpretations have been offered [65], [75], [76]. A second reason that organized sports participation may be misleading is that aspiring male athletes may be dismissed or “cut” from teams more often than their female counterparts or males may participate substantially more in unorganized settings, in organized sports programs that are unaffiliated with schools, or organized sports programs that are affiliated with colleges and universities but are below the intercollegiate level (e.g., intramural sports). Similarly, female participation in organized sports might be more likely to reflect extrinsic motives (e.g., obtaining a scholarship) than intrinsic interest. These possibilities have been suggested previously [77], [78], but the evidence offered for them was informal or based on unpublished studies.

### Aims

Here we tested whether, as our evolutionary hypothesis predicts, the modest sex difference in organized school sports participation in the U.S. substantially underestimates the sex difference in sports participation of all kinds (i.e., organized and unorganized; school affiliated and unaffiliated). By contrast, our evolutionary hypothesis predicts no reliable sex difference in non-competitive physical activity, which is hereafter called “exercise.” To test these predictions, we conducted three studies: Study 1 was based on time-use surveys; Study 2 was based on observations at public parks; Study 3 was based on intramural registrations at colleges and universities.

These studies also allowed us to test additional predictions of the evolved male predispositions hypothesis. One is that the sex difference in participation will be larger for team than individual sports. This prediction follows because team sports require both motivation to engage in physical competition and motivation to engage in cooperative group challenges. Both kinds of motivation are greater in males [28], [32], [37], [79]–[81]. However, individual sports require only the first kind of motivation, whereas team sports require both, suggesting that the sex difference should be larger for team sports.

Another prediction of the evolved male predispositions hypothesis is that the sex difference will remain stable over time. The logic here is that once genuine opportunities were consistently provided for organized female sports, usually by the 1980s or 1990s, female participation would have been limited by female interest. Female interest, in turn, would be determined by the interaction between innate predispositions (e.g., due to prenatal androgen exposure) and social influences that would be have been persistently relevant during human evolution history, such as peer interest and approval [82]. By contrast, informing girls that more collegiate scholarships will be available for them in 2010s than were available for their mothers in the early 1990s is not expected to affect their sports interest. This is because such a change is abstract, and it only applies to a small proportion of individuals.

We tested the prediction of historical stability in two ways. In Study 1, we addressed it indirectly, by comparing younger and older age groups, under the assumption that the sex difference in sports interest in a given cohort will remain stable. We addressed this prediction more directly in Study 3, by examining changes in undergraduate intramural registrations in the 2000s.

## Results

### Study 1: American Time Use Survey

Study 1 analyzed data from the American Time Use Survey (ATUS). The ATUS is a large, representative sample of all U.S. residents 15 years and older that was conducted continuously from 2003–2010 and included responses from 112,000 individuals [83]. Respondents reported all activities, including their durations, that they performed during the preceding 24-hour period.

Table 1 shows participation rates on a random day for each of 24 activities and for the summed activity types of individual sports, team sports, and exercise. As predicted, male participation rates for sports were significantly and substantially higher than female rates, especially for team sports. In particular, females comprised 28% of those who participated in individual sports and 20% of those who participated in team sports (ps <0.0001). There were significant sex differences in several sports, both individual and team, and in every case, males participated at higher rates. In contrast to sports participation, there was no substantial sex difference for total exercise participation, and, in fact, females actually comprised 51% of exercise participants. There were significant sex differences in several exercise activities, with some showing higher male rates (e.g., biking, weightlifting) and others showing higher female rates (e.g., walking, aerobics).

**Table 1:** Participation rates for sports and exercise activities on one day by males and females, American Time Use Survey 2003–2010.

Activity	Male		Female		% Female^b^
	%	CI	%	CI	
**Team Sports**					
Baseball	0.27	(0.19, 0.35)	NA	NA	NA
Basketball	1.33	(1.16, 1.49)	0.18	(0.12, 0.23)	12.5
Hockey	0.07	(0.04, 0.10)	NA	NA	NA
Football	0.48	(0.38, 0.57)	NA	NA	NA
Soccer	0.37	(0.29, 0.45)	0.12	(0.08, 0.16)	25.1
Softball^a^	0.15	(0.10, 0.20)	0.14	(0.10, 0.18)	49.8
Volleyball^a^	0.14	(0.09, 0.19)	0.17	(0.12, 0.21)	55.7
*Total Team Sports*	*2.69*	*(2.44, 2.93)*	*0.64*	*(0.56, 0.72)*	*20.2*
**Individual Sports**					
Bowling	0.32	(0.24, 0.39)	0.25	(0.20, 0.30)	45.5
Golf	1.08	(0.96, 1.20)	0.23	(0.18, 0.28)	18.4
Gymnastics^a^	NA	NA	NA	NA	NA
Racquet sports	0.37	(0.29, 0.44)	0.19	(0.15, 0.24)	36.1
Wrestling	0.05	(0.02, 0.08)	NA	NA	NA
*Total Individual Sports*	*1.81*	*(1.64, 1.97)*	*0.67*	*(0.59, 0.75)*	*28.2*
*Total Sports*	*4.45*	*(4.14, 4.76)*	*1.29*	*(1.18, 1.41)*	*23.7*
**Exercise**					
Aerobics	0.15	(0.11, 0.18)	0.60	(0.52, 0.68)	81.4
Biking	0.71	(0.62, 0.81)	0.32	(0.26, 0.38)	32.6
Dancing	0.22	(0.17, 0.27)	0.30	(0.24, 0.36)	59.0
Hiking^a^	0.15	(0.11, 0.19)	0.13	(0.09, 0.16)	46.7
Rollerblading	0.13	(0.08, 0.17)	0.05	(0.03, 0.08)	31.5
Running	1.47	(1.33, 1.61)	1.01	(0.90, 1.13)	42.4
Cardio	1.86	(1.69, 2.02)	2.13	(1.96, 2.30)	55.0
Walking	4.56	(4.31, 4.80)	5.34	(5.09, 5.58)	55.6
Water sports^a^	1.36	(1.23, 1.49)	1.40	(1.28, 1.52)	52.4
Weightlifting/strength training	2.71	(2.51, 2.92)	1.24	(1.11, 1.36)	32.7
Working out, unspecified	3.12	(2.90, 3.34)	2.77	(2.59, 2.96)	48.7
Yoga	0.11	(0.07, 0.16)	0.40	(0.33, 0.47)	79.0
*Total Exercise*	*13.9*	*(13.5, 14.3)*	*13.4*	*(13.1, 13.8)*	*50.7*
*Total Sports & Exercise*	*17.6*	*(17.1, 18.1)*	*14.5*	*(14.1, 14.9)*	*46.8*

CI = 95% confidence interval. p<.05 for difference between males and females except where indicated. NA = not applicable because (standard error/%) exceeds 0.30.

The exercise “caving, spelunking and climbing” is all NA (not shown).

a No significant difference between males and females.

b Estimates of % females do not correspond perfectly with male and female participation rates because there are more females than males in the population, especially in older age groups.

Sports and exercise participation rates varied with age (Table 2). The most striking pattern was that team sports participation was far higher in younger individuals: among those 15–19 years old, 17% of males and 5% of females participated, but for males over 60 and females over 50, rates were so low that they could not be reliably estimated. As predicted, despite this variability, the sex difference in participation for team and individual sports remained significant and substantial for all age groups. For team sports, the sex difference was smallest among those 25–29, with females comprising 23% of participants. For individual sports, the sex difference was smallest among those 15–19, with females comprising 33% of participants; females comprised 29–31% of participants in the other age groups. Exercise participation rates were comparatively high (i.e., >10% per day) for all age groups. Male rates were significantly higher than female rates among those 15–19, 20–24, 60–74 and 75+; female rates were significantly higher among those 30–39 and 40–49. Across all age groups, females comprised 41–55% of exercise participants.

**Table 2:** Participation rates for team sports, individual sports, total sports, and exercise on one day by age groups for males and females, American Time Use Survey 2003–2010.

Age Group		Team Sports	Individual Sports	Total Sports	Exercise
		%	%	%	%
	Male	17.0	2.57	19.4	15.8
15–19	Female	4.89	1.34	6.13	12.5
	% Female^b^	21.8	33.5	23.4	43.3
	Male	5.02	2.49	7.31	14.2
20–24	Female	NA	NA	1.53	10.3
	% Female	NA	NA	17.2	41.8
	Male	1.98	1.29	3.27	13.2
25–29	Female	0.60	NA	1.01	12.6
	% Female	23.0	NA	23.5	48.9^a^
	Male	1.46	1.26	2.71	12.1
30–39	Female	0.29	0.51	0.80	13.4
	% Female	17.1	29.4	23.3	53.1
	Male	0.75	1.38	2.12	11.9
40–49	Female	0.18	0.58	0.76	14.3
	% Female	19.5	30.5	27.1	55.5
	Male	0.25	1.63	1.88	13.9
50–59	Female	NA	0.63	0.69	13.8
	% Female	NA	29.0	27.9	51.2^a^
	Male	NA	2.49	2.68	16.3
60–74	Female	NA	0.94	0.95	14.2
	% Female	NA	30.1	28.7	49.8^b^
	Male	NA	2.23	2.23	18.3
75+	Female	NA	NA	NA	14.4
	% Female	NA	NA	NA	55.0

p<.05 for difference between males and females except where indicated.

NA = not applicable because (standard error/%) exceeds 0.30.

a No significant difference between males and females.

b Estimates of % females do not correspond perfectly with male and female participation rates because there are more females than males in the population, especially in older age groups.

A potential concern with measuring participation based on rates playing per day is that females might participate on fewer days but for longer durations. There were in fact significant sex differences in participation duration, but it was males, not females, who participated for longer durations. The sex difference in duration was 15% for team sports, 24% for individual sports, and 12% for exercise; these differences were significant (Table 3).

**Table 3:** Durations of sports and exercise for males and females, American Time Use Survey 2003–2010.

Sex	Team Sports^a^	Individual Sports^b^	Total Sports^b^	Exercise^b^
	Min/d	Min/d	Min/d	Min/d
Male	116.0 (75.2)	169.4 (96.5)	140.0 (89.4)	63.8 (52.4)
Female	101.3 (67.5)	136.4 (74.9)	122.2 (74.0)	56.8 (46.9)

Values indicate means and (standard deviations).

a p = .001;

b p<.0001.

Our predictions did not address educational achievement or race and ethnicity. Nevertheless, we explored whether these factors might have affected sports and exercise participation. Multivariate logistic regression indicated that non-whites, compared to Whites, participated more in team sports but less in individual sports; in addition, those who did not complete high school, compared to those graduated high school and those who obtained education beyond high school, participated more in team sports but less in individual sports (Table S1). Despite this variation, within each educational and ethnic group, substantial sex differences remained for both individual and team sports (Table S2). Among ethnic groups, the smallest sex difference for team sports occurred among Whites, where females comprised 25% of participants; the smallest sex difference for individual sports occurred among Blacks where females comprised 31% of participants. Among educational groups, the smallest sex differences occurred among college graduates; females comprised 30% of participants for individual sports and 28% for team sports.

### Study 2: Observations at Public Parks

Study 2 was based on systematic observations of unorganized sports and exercise participation at public parks in four U.S. locations: Grand Rapids, Michigan; State College, Pennsylvania; Tallahassee, Florida, and New Paltz, New York. Observations occurred in Summer and Fall 2011 and Spring 2012.

We documented a total of 2,879 sports and exercise participations (Table 4). Females accounted for a minority of participations for both sports (12%) and exercise (37%), although, as predicted, the sex difference was significantly greater for sports (χ^2^ (1, N = 2879) = 140.0, p<.0001). Also as predicted, the sex difference was significantly greater for team sports (10% female) than individual sports (19% female) (χ^2^ (1, N = 2552) = 29.7, p<.0001). The sex difference was significant for all frequently occurring sports (Table 4). By contrast, there was no significant sex difference for popular exercise activities, with the exception of skateboarding, which was done by mainly by males (Table 4).

**Table 4:** Sports and exercise participations at public parks by males and females.

Activity	Male	Female	% Female
**Team Sports**			
Baseball	95	11	10.4
Basketball	685	51	6.9
Football	267	13	4.6
Soccer	445	56	11.2
Softball	64	16	20.0
Ultimate frisbee	116	31	21.1
Others	88	23	20.7
*Total Team Sports*	1760	201	10.2
**Individual Sports**			
Disc golf	127	2	1.6
Tennis	351	86	19.7
Others	3	22	88.0
*Total Individual Sports*	481	110	18.6
*Total Sport*	2241	311	12.2
**Exercise**			
Biking	23	21	47.7^a^
Running	34	36	51.4^a^
Skateboarding	63	2	3.1
Walking	62	56	47.5^a^
Other	24	6	20.0
*Total Exercise*	206	121	37.0
*Total Sports and Exercise*	2447	432	15.0

p<.01 for difference between males and females except where indicated.

a No significant difference between males and females.

Participations may not reflect independent decisions to participate because people may plan to meet at a park or an individual might become more likely to participate after observing others doing so. Thus, a key question is whether the sex difference in sports will remain strong if we examine the number of groups or parties, rather than individuals. For individual sports, there were 216 parties, and 7% were female only, 2% were female biased (more females than males), 16% were unbiased, 5% were male biased, and 70% were male only. For team sports, there were 389 parties, and 3% were female only, 2% were female biased, 5% were unbiased, 15% were male biased, and 74% were male only. Thus, a large majority of sports parties were comprised of more males than females, both for individual sports (75%; binomial test, p<.0001) and team sports (89%; p<.0001).

A related issue is the extent to which the sex difference might be due to males being more comfortable playing in larger groups (see [36], [37], [80], [84]). We therefore tested whether percentage female participation decreased as party size increased. In fact, female percentage of participation was uncorrelated with party size, for both individual sports (r(214) = .04, p = .57) and team sports (r(387) = −.04, p = .38). Thus, females were a consistently small fraction of participants, in parties ranging from one (10% of 52 participants) to parties of six or more (11% of 1,343 participants). One consequence of this pattern was that large groups comprised entirely of females were rare. Specifically, there were only two all-female groups with six or more individuals, whereas there were 87 all-male groups of this size.

Observations were made by seven researchers at 41 parks, allowing an examination of the consistency of sex differences across locations and researchers. Some parks clearly fostered particular activities. Most notably, 85% of skateboarding participations occurred in one park, and 89% of disc golf participations in another park. Nonetheless, many sports, especially the popular team sports of soccer and basketball, occurred in many parks, and the sex difference generally occurred reliably. Most crucially, all seven researchers documented at least 108 team sports participations, and all found a large, significant sex difference (Table 5). Moreover, the four male observers recorded essentially the same percentage of female participants in team sports (M = 11.9) as the three female observers (M = 11.6; t (5) = .18, p = .91). Similarly, the two researchers (one male, one female) who were blind to the hypothesis of a sex difference recorded a percentage of female participants in team sports (M = 12.6) that did not differ from that documented by the other researchers (M = 11.4.; t(5) = .42, p = .69).

**Table 5:** Sports and exercise participations at public parks by researcher for males and females.

State	Researcher	# Parks	Exercise			Individual Sports			Team Sports		
			Male	Female	% Female	Male	Female	% Female	Male	Female	% Female
MI	ROD	9	34	20	37.0^a^	81	17	17.3	559	43	7.1
MI	EF	12	31	15	32.6^a^	12	19	61.3^a^	450	43	8.7
MI	DB	4	65	2	3.0	18	1	5.3	94	14	13.0
PA	JK	7	2	1	33.3^a^	82	4	4.7	204	26	11.3
PA	CM	6	54	68	55.7^a^	92	29	24.0	192	31	13.9
FL	BW	3	0	0	NA	191	39	17.0	145	28	16.2
NY	TG	1	20	15	42.9^a^	5	1	16.7^a^	116	16	12.1

p<.01 for difference between males and females except where indicated.

a No significant difference between males and females.

Researchers could only broadly estimate participants’ ages. Nonetheless, these estimates indicated that the sex differences (Table 4) held across age groups, at least for age groups with many observations (Table 6).

**Table 6:** Sports and exercise participations at public parks by age groups for males and females.

Age (yrs)	Exercise			Individual Sports			Team Sports		
	Male	Female	% Female	Male	Female	% Female	Male	Female	% Female
0–12	41	15	26.8	26	30	53.6^a^	305	35	10.3
13–19	84	23	21.5	113	40	26.1	575	61	9.6
20–49	66	58	46.8^a^	315	36	10.3	851	81	8.7
50+	15	25	62.5^a^	27	4	12.9	29	24	45.3^a^

p<.01 for difference between males and females except where indicated.

a No significant difference between males and females.

### Study 3: Intramurals at Colleges and Universities

Study 3 involved surveys of intramural sports registrations at colleges and universities in the U.S. An intramural sport is generally played by an undergraduate, usually between 18 and 24 years of age, who does not play the sport at the intercollegiate varsity level. There are generally no extrinsic incentives (e.g., substantial prizes or publicity) for intramurals, so they should provide a reliable indicator of intrinsic motivation to participate. Most intramural registrations entail playing a series of games (e.g., a six game season occurring over an academic semester). Data were from 2010 and 2011.

Thirty-four institutions provided information (see Methods) on total male and female intramural registrations, and females accounted for 26% of total registrations across all institutions and sports. This pattern held across institutions, as the median value was 28%, and the sex difference was significant at every institution (Table 7). In fact, there was not a single institution where females reached 43% of registrations, the female percentage of participation across all NCAA intercollegiate sports [62].

**Table 7:** Sex differences in enrollments and intramural (IM) sports participations.

Institution	Undergrads	% Female Undergrads	IM Registrations	% Female IM Registrations
Alabama	24882	52	10903	19
Arkansas St.	10051	58	3577	24
Arkansas Tech	9138	52	2787	33
Bloomsburg (PA)	9136	57	4544	34
Boise St. (ID)	17349	54	2635	28
Boston College (MA)	9895	52	8879	20
California University (PA)	7419	52	1802	21
Central Missouri	9168	54	4868	35
Central Washington	11052	50	3119	25
Cincinnati (OH)	22449	51	5193	25
Connecticut	17345	49	13903	30
Duke (NC)	6697	49	6734	20
Eastern Michigan	18554	57	1594	29
Emporia St. (KS)	4066	61	1177	31
Fort Lewis (CO)	3853	49	1489	42
Grand Valley St. (MI)	20986	58	7190	31
Lenoir-Ryne (NC)	1570	62	247	13
Minn. St. Moorhead	6997	57	790	32
Nebraska-Kearney	5162	53	4865	38
Northwestern (IL)	9535	52	10106	23
Ohio Univ.	20994	57	6961	28
Oregon St.	19557	47	11429	28
Pittsburg St. (KS)	5891	46	3003	24
Shippensburg (PA)	7143	52	2041	30
SMU (TX)	61938	53	4792	25
Stanford (CA)	6940	49	6319	18
Stonehill (MA)	2582	62	1988	37
Texas A&M	39148	47	23121	24
Texas Tech	25462	45	14210	26
Toledo (OH)	18130	50	15673	23
UAB (AL)	11028	58	4972	28
UNLV (NV)	22534	55	4546	28
Washington St.	21816	52	13358	28
Wingate (NC)	1622	54	1056	26

p<.0001 for difference between males and females at all institutions.

Twenty-seven institutions provided information regarding registrations for specific sports (Table 8). Fewer than 4% of registrations were for individual sports, the most popular of which were tennis (0.8% of all specific registrations), bowling (0.7%), running (0.5%), golf (0.4%), and racquetball (0.3%). Across these 27 institutions, females accounted for 31% of individual sports registrations, whereas they accounted for 26% of team sports registrations, a significant difference (χ^2^ (1, N = 175697) = 68.1, p<.0001). The greater sex difference in team than in individual sports occurred at 13 of the 23 institutions where individual sports occurred, and this difference was significant in nine cases (p<.05 for each). Of the 10 institutions where, contrary to our hypothesis, the sex difference was greater in individual than in team sports, the difference was significant in five cases.

**Table 8:** Percentage female participation for team, individual, and popular intramural sports.

Institution	Team	Individual	Football	Soccer	Basketball	Softball	Volleyball
Alabama	19	18	16	20	15	26	55^a^
Arkansas St.	22	37^a^	25	15	23	26	27
Arkansas Tech	34	24	22	47^a^	33	36	49^a^
Bloomsburg (PA)	34	NA	0	30	22	50^a^	51^a^
Boise St. (ID)	26	19	6	38	18	33	52^a^
Boston College (MA)	20	29	4	24	14	23	48^a^
Central Missouri	35	31	25	34	27	28	60
Cincinnati (OH)	25	20	17	28	17	27	48^a^
Connecticut	30	33	26	34	22	20	42
Eastern Michigan	28	44^a^	19	35	15	NA	52^a^
Emporia St. (KS)	31	38^a^	20	33	21	38	49^a^
Grand Valley St. (MI)	30	42	15	34	20	32	53
Lenoir-Ryne (NC)	13	NA	0	19	20	8	NA
Minn. St. Moorhead	32	33^a^	6	15	27	NA	56^a^
Nebraska-Kearney	39	22	30	28	32	49^a^	58
Northwestern (IL)	23	NA	18	23	15	21	30
Ohio Univ.	28	28	17	30	21	27	52^a^
Oregon St.	27	34	16	33	21	31	50^a^
SMU (TX)	25	38^a^	28	26	14	17	38
Stanford (CA)	17	26	11	14	9	16	31
Stonehill (MA)	37	43^a^	0	49^a^	22	25	59
Texas A&M	24	21	15	30	14	18	53
Texas Tech	26	NA	13	23	23	26	38
UAB (AL)	27	36	27	21	23	27	57^a^
UNLV (NV)	29	8	29	33	21	35	39
Washington St.	27	51^a^	17	32	18	27	58
Wingate (NC)	27	13	27	33	19	NA	37^a^

p<.01 for difference between males and females except where indicated.

a No significant difference between males and females.

The most popular team sports were football (20% of all specific registrations), soccer (20%), basketball (19%), softball (16%), and volleyball (13%). Females did not account for the majority of registrations in any of these popular sports, although they came close in volleyball (football: 16%; soccer: 29%; basketball: 20%; softball: 27%; volleyball: 48%). These patterns were fairly consistent across institutions, with males being a significant majority of registrants at most institutions for all popular sports, with the exception of volleyball (Table 8).

An interesting question is whether female sports participation is depressed due to the presence of male competitors. Intramural data are useful for addressing this because, at most institutions, team sports mainly involve single-sex competition (M = 71% of teams were single-sex rather than co-ed; SD = 5.5%; n = 10 institutions). Eight institutions provided data on single-sex registration for popular sports. In this context, females comprised a modest percentage of participants (football: 20% of single-sex registrations by females; soccer: 24%; basketball: 20%; softball: 15%; volleyball: 45%).

Intramural registrations generally involve playing in a series of games over a semester. It is therefore possible that registration might underestimate relative female participation because, for any given registration, females might participate more frequently. Five institutions provided information on participations per registration separately for males and females. In all five cases, however, males participated at greater rates (University of Toledo: 11% greater male participation per registration; University of Nevada, Las Vegas: 17%; Boise State University: 22%; Fort Lewis College: 44%; Southern Methodist University: 98%). In fact, if the median difference in participation rate (i.e., 22%) is extrapolated to all 34 institutions in Table 7, it suggests that females account for 26% of intramural registrations but only 22% of intramural participations.

Another concern is that perhaps there are more males than females at the institutions in our sample, and this might be partly responsible for the large sex difference. However, across all institutions, females comprised 52% of undergraduates, and there were more female than male undergraduates at 24 of 34 institutions (Table 7). This pattern further indicates that the sex difference in registrations underestimates the sex difference in individual participation rates.

A crucial question is whether the sex difference in sports participation is decreasing historically. We were able to address this question because nine institutions provided at least five years of intramural data, some on total male and female participations and others on unique male and female participations (i.e., each individual counts once regardless of how many sports they play over the entire academic year). As shown in Table 9, at all nine institutions, the percentage of female intramural registration was similar in the last year of available data, usually 2010–2011, as it was in the preceding years. By this measure, seven institutions showed decreasing female registration and two showed increasing female registration. Similarly, correlations between year and percentage of female registration were negative at five institutions and positive at four institutions; none was significant, perhaps due to small size (Table 9). To increase statistical power, we used a general linear model to incorporate data from all institutions: percent female registration was significantly predicted by institution (F (8,55) = 21.6; p<.0001) but not by year (F (1,55) = 0.57; p = .45). In fact, the parameter estimate for year was negative (β = -.06), indicating a very slight decrease in female registration. We also repeated this analysis weighting each institution’s data by its mean number of total registrants per year. In this case, the parameter estimate for year was positive, yet it was still slight (β = .07).

**Table 9:** Percentage female participation in intramural sports over time.

Institution	Years	All years	Final year	Correlation
Arkansas St.	2004–2010	24.0	24.3	0.37
Arkansas Tech	2001–2005	30.2	29.6	−0.71
Boise St.(ID)	1997–2010	29.4	27.8	−0.07
Connecticut	2006–2010	28.9	30.3	0.67
Grand Valley St.(MI)	2004–2010	37.4	36.2	−0.48
Minnesota St. Moorehead	2001, 2003–2008, 2010	36.7	33.9	−0.46
Northwestern (IL)	2006–2010	23.7	22.7	−0.42
Stonehill (MA)	2006–2010	37.6	37.3	0.39
Texas Tech	2002–2010	26.2	25.1	0.50

No correlation reached statistical significance, p<.05.

## Discussion

On the basis of an evolutionary history of one-on-one and coalitional competition among males, we hypothesized that men and boys possess an evolved predisposition to be interested in competitive physical activities, including sports. That males have apparently participated and monitored sports more often than females in most or all societies supports this hypothesis, but surging female participation in organized school sports in the contemporary U.S. challenges it. The three studies reported here, however, demonstrate that organized school sports participation substantially underestimates the sex difference. Specifically, females comprise approximately 42% of high school athletes and 43% of collegiate athletes, but they comprise only 24% of those who report playing sports on a given day (Study 1), 12% of those playing sports in public parks (Study 2), and 26% of those who register for collegiate intramural sports (Study 3). Even these percentages somewhat underestimate the sex difference because males play for longer durations (Study 1) and play more frequently per intramural registration (Study 3).

In addition, as predicted from our evolutionary framework, the sex difference in sports participation was greater for team than individual sports. This result was clear in Study 1 and Study 2. Study 3 also found a greater sex difference for team than for individual sports, although the difference was not consistent across institutions, perhaps owing to the fact that intramurals at most institutions rarely involve individual sports.

In contrast to both individual and team sports, the sex difference in exercise was unreliable (Study 1) or modest (Study 2). The exercise results indicate that both males and females are motivated to be physically active, but that males are generally more interested in pursuing this in a competitive way (see [85]–[89]).

Although our findings contradict the popular claim that there is no substantial sex difference in sports interest [63]–[65], they are in agreement with previous empirical studies of sports participation. Whether measuring duration or frequency, these studies consistently find that males play sports, especially team sports, at least twice as much females do, and often much more frequently. This is the case with children in Australia [90], teens in Canada [91], children in Denmark [92], teens and adults in England [44], and children, teens and adults in Ireland [43].

Also paralleling the present study, when studies report non-competitive physical activity or exercise, they indicate minimal sex differences [43], [44], [90]. Other studies that measured overall physical activity (sports and exercise combined) in large societies also report modest differences [93], [94], sometimes with females participating more [95]. Finally, earlier studies of sports participation among children and teens in the U.S. (with modest, non-representative samples) reported that males play sports more than twice as often as females [96], [97].

### Potential Limitations

Each of our three studies has potential limitations, yet none seriously challenge our conclusions. One possible limitation of Study 1 is that self-reports of physical activity typically show only low to moderate criterion validity, especially when based on retrospective queries [98]. However, the methods used in time use surveys, such as the ATUS, have been specifically designed to minimize distortions [83]. More importantly, we obtained converging results in Study 2 and Study 3, and they were based on behavioral measures. A second potential limitation of Study 1 is that the size of the age group cohorts was modest given daily sports participation rates. For example, in the 20–24 age group, there were 5,189 respondents, and the participation rates indicate that only about 460 individuals would have reported playing sports on the previous day. However, there was a highly similar sex difference in Study 3, which included over 500,000 intramural registrations.

Another limitation of Study 1 is that, because there were no ATUS codes for them, we could not include the high school sports of water polo, lacrosse, swimming and diving, or competitive cheerleading [61]. Water polo and lacrosse have greater male than female participation (water polo: 47% female participation; lacrosse: 43%) and were probably not included in the ATUS because they are played much less than most other sports (see Methods for sports popularity). Thus, their inclusion seems unlikely to have substantially affected our results. Swimming and diving, however, is a moderately popular sport and females constitute 55% of participants. Therefore, the sex difference in individual sport participation in the present study is likely to be somewhat of an overestimate. Finally, competitive cheerleading is almost exclusively done by females (98%), and is moderately popular. However, although it meets our technical definition of a sport, competitive cheerleading is unusual because it is the only popular “genuine” team sport (see Methods) whose outcome depends exclusively on judging, not direct competition between simultaneously competing teams. Moreover, it is the only major high school sport that is not an NCAA intercollegiate sport. Thus, although including competitive cheerleading would somewhat decrease the sex difference in team sports participation, it is not clear that including it is desirable, at least with regards to testing the evolved predispositions hypothesis.

Study 2 was potentially limited by the fact it was based on convenience sampling, meaning that is possible that other, more representative public parks would not show a pronounced sex difference in sports participation. For several reasons, though, this possibility seems highly unlikely. First, we know of no bias in our sample, and all seven researchers independently documented a large sex difference in team sports participation. Indeed, we are unaware of any credible report of an area in the U.S. where females consistently play sports in public areas at rates similar to males. Second, Study 1 was based on a nationally representative sample, and it indicated the sex difference occurred for all educational and ethnic groups. Similarly, Study 3 found the sex difference across a broad sample of colleges and universities.

Study 3 might be viewed as limited because it focused on intramural sports, which are played mainly by undergraduates in their late teens and early 20s. In fact, this was an ideal sample for rigorously testing our main prediction. As revealed in Study 1, most sports, especially team sports, are played by those 24 years and younger. In addition, because participation rates in organized school sports are roughly 18 times greater in high school than in college [99], it might be more difficult to assess intrinsic motivation in high school students because much of their sports participation might be linked to preparing for organized school competition. Furthermore, women seeking college degrees might be expected to show greater sports interest than other women because there apparently are reciprocal relationships between education and sports [100]–[102].

### Participation and Interest

The evolved male predispositions hypothesis assumes that the large sex difference in sports participation reflects a large sex difference in sports interest. Is this assumption valid? It is a truism that many individuals with a strong interest in sports (or other activities) might not participate owing to lack of time, access to facilities, or other constraints. Although constraints on females’ sports participation must be greater than the constraints on males in some cases, for at least three reasons, this seems inadequate as a general explanation for females’ lower participation.

First, although females, especially girls, may have less time for sports and leisure in most societies [103], lack of time is not a plausible explanation for our results. The reason is that in Study 1 females spent nearly as much time as males in total physical activity; the key difference was that the proportion of time females spent on sports, especially team sports, was much less. In particular, for males 15% of their physical activities involved team sports and 10% involved individual sports; for females 4% involved team sports and 5% involved individual sports. Other studies also indicate that the sex difference in sports participation partly reflects females giving higher priority to other recreational and extracurricular activities [104], [105].

Second, that females do not play sports as much as males because they lack facilities or opportunities also seems unlikely, at least as a general explanation. This is illustrated by soccer and basketball, the two most frequently played team sports in the U.S. These sports require minimal equipment and facilities, and on school teams, female participation is almost as high as male participation [61], [62]. Yet all three studies reported here find that males play these sports more than three times as much as females do. This pattern even held for single-sex collegiate intramurals, showing that females’ relatively low participation cannot be ascribed to the presence of male competitors. Finally, if constraints were a major factor, then females’ self-reported desire to participate and excel in sports would be equal to males’, and studies consistently contradict this [67]–[70].

### Temporal Convergence

The present findings, together with questionnaire studies, demonstrate that there is a substantial sex difference in sports interest even in the contemporary U.S., a society where there is consensus that great progress has been made in equalizing organized school sports opportunities [63]–[65], [106]. Nonetheless, proponents of the “no intrinsic differences” view might contend that it has been only 40 years since Title IX was passed, and that this legislation was not effectively implemented during some of these years [64]. Thus, although there is a large sex difference now in sports interests, this difference might be waning.

Although the time depth was limited, Study 3′s analysis of temporal changes in intramural participation at nine institutions does not support the claim of convergence. Similarly, Study 1 compared older and younger groups with the ATUS and found no indication that younger women, who grew up with Title IX being better enforced, participate at relatively greater rates. Testing for changes over time with the ATUS would also be valuable, but we could not do so here because preliminary analyses indicated that, because of modest sample sizes, yearly sports participation estimates were highly unstable.

Another set of studies addressed historical convergence in a less direct fashion, focusing on sex differences in willingness to train competitively in distance running. Deaner [85], [107], [108] showed that, although the number females that participate in distance running in the U.S. has grown steadily since the 1970s, so that there is no longer a sex difference in participation, there are still roughly three times as many males that run fast relative to sex-specific world class standards. For example, in a typical local 5 K road race with equal male and female participation, for every female that finishes within 25% of the female world record, there are roughly three males that finish within 25% of the male world record. This pattern holds robustly for elite runners and non-elite (i.e., recreational) runners, and tests reveal no indication that the sex difference in the number of relatively fast performers has diminished over the past few decades. Because relative running performance is an equally strong predictor of training volume (e.g., kilometers/week) in men and women [109], these patterns indicate that the sex difference in willingness to train competitively is large and stable [85], [107], [108]. Apparently, the large increase in female runners has mainly involved those who run for non-competitive reasons; most competitive females were already competing by the 1980s or early 1990s.

We hope that other measures of sports participation and interest can be identified that will allow assessments of temporal change. Similarly, it should be possible to revisit this issue in the future, after Title IX has had another generation or two to take effect. Nonetheless, for now, the hypothesis that the sex difference in sports interest is in the process of converging must be viewed as lacking empirical support.

### Alternative Explanations

Another argument that might be raised in defense of the “no intrinsic differences” view is that the changes produced by Title IX–changes focused on opportunities and incentives in organized school sports–are insufficient to catalyze female sports interest. For example, compared to boys, girls may still receive less childhood sports encouragement [110] or lack role models of professional athletes (see [111], [112]). Although these and related hypotheses certainly warrant attention, it is difficult to conceive of practical methods for assessing them, particularly because individuals and their environments interact. In the case of sports encouragement, for instance, parents may be less likely to enroll their daughters in soccer leagues, but this may reflect that fewer young girls than boys express interest in this sort of activity [36], [113].

Similarly, the fact that there are far fewer women than men who earn their livelihoods playing sports can be viewed as an effect, rather than a cause, of lesser female sports interest. For example, the premier men’s basketball league in the U.S., the National Basketball Association (NBA), has sponsored a women’s professional league (WNBA) since 1997, and the attendance and viewership is a small fraction of the NBA’s and has not grown [114]. Similarly, in the late 1990s a magazine was launched called Sports Illustrated Women (SI Women). SI Women was targeted to appeal to girls and women who wanted follow high-level women’s sports in the way that Sports Illustrated caters to the interests of male sports fans. However, publication of SI Women ceased in 2002 because there was not a market to support it [115], [116]. Other magazines focusing on elite female athletes have also failed to gain large readerships [115].

Finally, in considering the argument that Title IX might not be sufficient to substantially spur female sports interest, it is worth noting that this argument contradicts the claims of many scholars and the U.S. courts. The courts, in particular, have been clear that one of Title IX’s main purposes is to produce equal sports opportunities in schools and that doing this will, by itself, be sufficient to eventually produce equal sports interest [63]–[65].

### Conclusions

Our findings support the hypothesis of an evolved male predisposition for physical competition–one that manifests in contemporary societies as greater participation of males in sports. Two points about this hypothesis must be stressed, however. First, as noted in the Introduction, we do not claim that sports interest is entirely driven by an evolutionary history of male-male competition and the proximate effects of sex hormones. On the contrary, sports interest is influenced by societal gender roles, parents, peers, and the like, and such factors likely contribute to variation across individuals and societies [48]–[50], [53]–[55], [110]. In fact, as previous scholars have shown, evolutionary theory is fully compatible with substantial cross-societal variation in human sex differences [32], [59], [117]–[119], and sports participation is no exception. Nonetheless, the evolved male predispositions hypothesis does predict that males will, on average, be more interested in physical competition, and thus sports, in all or nearly all societies. A recent study illustrated these points: it found more male than female sports in all societies in the Human Relations Area Files probability sample, yet the sex difference was typically greater in patriarchal than in non-patriarchal societies [40].

Our final point is that a greater male predisposition for sports interest does not contradict most arguments made by Title IX proponents. Most notably, it is indisputable that, prior to Title IX, girls and women in the U.S. generally had vastly inferior sports resources and opportunities than boys and men, that sports and exercise can provide substantial benefits for girls and women, that strong moral arguments exist for ensuring that males and females enjoy equal sporting opportunities, and that Title IX has had many positive effects. Nevertheless, our results do suggest that it may be a mistake to base Title IX implementation on the assumption that males and females have, or soon will have, generally equal sports interest.

## Materials and Methods

### Study 1

The American Time Use Survey (ATUS) is a survey of the use of time among the civilian, non-institutionalized population aged 15 years and older conducted by the U.S. Bureau of Labor Statistics and the U.S Census Bureau [83]. The sample represents a stratified random subsample drawn from a panel of households that completed participation in the Current Population Survey (CPS), a federal survey that provides the national unemployment rate. A single person from each household that was selected from the CPS panel was interviewed by telephone about use of personal time on a single, pre-assigned reporting day. Interviewers asked respondents to report all activities that were performed during the 24-hour period beginning at 4∶00 am on the day before the interview and ending at 3∶59 am on the day of the interview. Respondents were given the opportunity to report spontaneously recalled activities, the times of day that activities started and ended or one time of day and how long that activity lasted. Verbatim responses to activities reported in the interview were later coded by two interviewers into >400 categories including 25 sports and exercise activities described below. Households without telephones were encouraged to respond by mailing them a $40 debit card that could be activated if they called in to complete the survey. The ATUS is a free public use data set [120].

Annual sample sizes ranged from 12,248 in 2007 to 20,720 in 2003, though after 2003 the target was 13,000 completed interviews [83]. Response rates of persons selected from the CPS panel ranged from a low of 52.5% in 2007 to a high of 57.8% in 2003. For the current study, we analyzed data from all 112,038 persons aged 15 years and older who were surveyed between 2003 and 2010. In total, the ATUS interviewed 48,687 males and 63,351 females aged 15 to 99 years, including 7,624 15- to 19-year-olds (3,753 females and 3,871 males) and 5,189 adults aged 20 to 24 years (2,953 women, 2,236 men). Seventy percent were White, 13% were Black, 13% were Hispanic or Latino and 4% were other race or multi-racial. Thirty percent were college graduates, 17% had less than high school education, and the rest were high school graduates or had some college or technical school education.

We focused on sports with moderate to high participation rates in U.S. high schools. These were defined as sports that were played by more than 20,000 total girls and boys in 2009 [61]. These sports are: baseball (about 473,000 or 473 K participants), basketball (980 K), bowling (53 K), competitive cheerleading (126 K), cross country (442 K), field hockey (64 K), (American) football (1132 K), golf (229 K), gymnastics (21 K), ice hockey (45 K), lacrosse (159 K), soccer (748 K), softball (394 K), swimming and diving (290 K), tennis (345 K), track and field (1169 K), volleyball (454 K), water polo (39 K), and wrestling (279 K). We focused on high school sports rather than collegiate sports because high school sports participation is roughly 18 times greater [99].

Unfortunately, the ATUS lexicon did not correspond with the high school sports in several ways. First, there were no ATUS codes for swimming and diving, water polo, lacrosse, or competitive cheerleading [121], so these sports could not be assessed. Second, the ATUS had no code for tennis, although it did have a code for racquet sports, which we used because it encompasses and largely consists of tennis. Third, the ATUS code for hockey did not distinguish ice hockey and field hockey, so we used that code and assessed both kinds of hockey together. Fourth, we used the ATUS code for running because there were no codes for cross-country or track and field.

We defined exercise activities as ones apparently undertaken primarily for physical fitness rather than competition. We selected these based on their availability in the ATUS lexicon [121] and their popularity in the U.S. [93]. Exercise activities were aerobics, biking, dancing, hiking, rollerblading, running, cardio, walking, water sports, weightlifting/strength training, working out (unspecified), and yoga. We classified running as an exercise rather than a sport because studies of distance running find that most runners’ self-reported motivation and training is consistent with a non-competitive orientation [89], [122]. Although respondents could self-report participating in “caving, spelunking or climbing,” the prevalence of this exercise category was too low to report separately for males and females.

We classified sports as individual sports or team sports. We classified bowling, golf, gymnastics, racquet sports, and wrestling as individual sports despite the fact that these sports can involve team competition (and generally do in U.S. high schools). We did so because an individual’s performance in these sports depends almost on entirely on their own efforts, rather than coordinated efforts with their teammates, a point revealed by the fact that these sports invariably include individual championships. By contrast, in “genuine” team sports, individuals may garner awards (e.g., “all star”), but there are no individual championships. We classified baseball, basketball, hockey, football, soccer, softball, and volleyball as team sports.

ATUS respondents were classified as having (or not having) participated in an activity on the recalled day for each of the 25 identified activities, for each of the three groups of activities (i.e., team sports, individual sports, and exercise), and for either individual or team sports. In addition, among those who participated in an activity group, we obtained their total minutes of participation.

We used tests of equality of proportions to assess the statistical significance of sex differences. We used multivariate logistic regressions to assess the effects of demographic characteristics on participation in team and individual sports. All analyses were weighted and, for the prevalence estimates, confidence intervals were computed from estimates of total variance according to methods used for the Current Population Survey [123]. For analysis, we used SAS version 9.1 (Cary, NC, 2003).

### Study 2

#### Ethics statement

This study [243297-1] was approved as “exempt” by Grand Valley State’s Institutional Review Board (IRB) on June 6, 2011.

Each researcher was instructed to initially identify public parks where unorganized sports were often played. Each park was required to have at least one of the following: basketball court, tennis court, grass field or turf field. Parks might include other facilities such as a running track, handball courts, horseshoe pit, disc golf course, or skateboarding ramp. Researchers were instructed to avoid parks with pools, lakes, or other areas allowing aquatic sports. Because this study focused on sports, not exercise, researchers were also instructed to avoid trails where people walk, run, bike, or rollerblade. Parks often included distinctive areas for potential sports play (e.g., basketball court, softball diamond); these areas were considered part of the same park so as long as the researcher could visually monitor all areas simultaneously and there was no street dividing the areas. Parks could include the grounds of public schools so long as the schools were not in session. University gyms, sports clubs, and other non-public areas were not included.

Parks were selected based on the apparent occurrence of sports and researchers’ convenience in visiting them, which usually meant they were in the same geographical area. Each researcher was asked to identify a “circuit” of five to twelve parks, although sometimes circuits were smaller due to a researcher’s transportation limitations or because there were few local parks. Researchers’ circuits generally did not include common parks, although EF made twelve observations (45 participations) at one of ROD’s main parks (194 participations). Once researchers began observations, they did not add parks to their circuits, although they stopped visiting parks where they repeatedly observed no sports. The locations of the park circuits were fairly diverse: in Grand Rapids, Michigan, two circuits (RD’s, EF’s) occurred within the city, whereas another occurred in suburban towns west of the city (DB’s); in State College, Pennsylvania, a college town, the two circuits occurred within the city; in New Paltz, New York, a small college town, the circuit consisted of the single suitable public park; in Tallahassee, Florida, the circuit occurred within the city. Observers were aware of the sex difference hypothesis with the exception of the two observers in Pennsylvania who neither knew nor suspected that this study was focused on sex differences.

At times when sports participation seemed likely, such as early evenings or weekends when the weather was good, researchers would deliberately visit all parks in their circuit. To avoid bias, researchers did not make observations opportunistically, such as upon noticing sports being played when they were driving by a park. Researchers often completed their circuit several times per week but not more than once per day. No attempt was made to avoid repeated observation of the same individuals on different days. This could not be done reliably. Furthermore, participation frequency is actually a good measure for addressing the hypotheses of interest.

Upon arrival at a park, the researcher would document all instances of exercise or sports that were currently occurring (i.e., instantaneous time sampling [124]). Activities were counted as occurring if individuals were taking a brief recess related to the activity (e.g., choosing teams, drinking fluids, tying shoes). However, in such cases, individuals were counted as participating only if they resumed participation with three minutes of the researcher’s arrival. To avoid bias, researchers did not wait at parks for activities to be initiated.

We classified activities as sports or exercise based on Study 1. Because of their similarities with other sports and because they met our definition, the following activities were also classified as sports: ultimate frisbee (147 total participations), disc golf (129), muggle quidditch (24), horseshoes (22), lacrosse (19), wiffleball (17), and kickball (8). The following were also classified as exercise: skateboarding (65), hackeysack (12), riding scooters (6), non-combative martial arts (4), and hula-hooping (1). We classified sports as individual sports or team sports based on Study 1.

Some instances of sport participation involved practice rather than competition. Examples include playing catch with a baseball or football, practicing shooting in basketball or soccer, or hitting a tennis ball against a backboard. We classified this kind of activity as sports participation, on the assumption that it is generally undertaken to improve one’s ability to compete in a sport.

We classified instances of sports participation as organized or unorganized. We defined organized sports as those that are directed by individuals besides the participants. Examples include high school sports, collegiate sports, club sports, intramural sports, recreational leagues, and training sessions organized by coaches. Researchers did not interact with participants and so did not ask sports participants if there was an organizing body or agent directing their play. Instead, researchers categorized participation as organized if they saw evidence of any of the following: uniforms, referees, judges, coaches, or formal leadership (e.g., team captains directing practice). We did not present data on organized participation in the Results section because there were relatively few organized sports parties observed (83) but most were large (M = 19.4 individuals), meaning that random error could substantially affect the results. Overall, females accounted for 25% of organized sports participants.

The data collection protocol was designed to promote reliability. ROD and EF tested this by simultaneously and independently collecting data on two evenings. Reliability was high for sex (Cohen’s κ = .99; n = 169), age group (κ = .95; n = 169), party exercise/activity classification (κ = .90; n = 22), and party organized/unorganized sport classification (κ = .90; n = 22).

### Study 3

#### Ethics statement

This study involved surveying intramural organizers/administrators at colleges and universities. They voluntarily reported institutional data about demographic patterns of participation at their institution. They provided no data regarding individuals, meaning that this study did not technically include “human subjects” and thus did not require IRB approval.

We requested data from institutions that play football in the NCAA Division I (D1) Football Bowl Subdivision (FBS) or in NCAA Division 2 (D2). We searched the website of each institution for an individual who was identified the primary intramural organizer. We identified such an individual at 74 of 120 D1 institutions and 73 of 151 D2 institutions. We then contacted these individuals by email; for D1 institutions, we did this in late October and early November 2011; for D2 institutions we did this in March 2012. We explained that we were conducting a study of intramural sports participation to assess which sports are most popular for men and women in different regions of the United States. We asked which sports were offered as intramurals at their institution, how many men and women registered or participated in each sport and in all sports combined, and whether each sport was offered as co-ed, single sex, or both. We requested data regarding the past year and any previous years. If we received no response, we sent one additional request about one week later.

Of the 74 D1 institutions, 36 responded but several did not provide useful data (e.g., they did not distinguish male and female registration); 19 provided data on overall sex differences in registration of all kinds, single sex and co-ed together (Table 7), five institutions provided data on male and female single-sex registrations (University of Louisville, University of Mississippi, University of Notre Dame, Wake Forest University, Western Michigan University); University of Nevada, Las Vegas provided both kinds of information. Of the 73 D2 institutions, 29 responded; 15 provided data on overall sex differences in registration of all kinds (Table 7); Millersville University provided data on male and female single-sex registrations; Shippensburg University provided data on both.

Our primary measure of participation was registration, and one individual could register for several sports each year. If we had focused on unique participants, the sex difference would have been substantially smaller. However, total participations was the appropriate measure for the current study, which aimed to measure participation frequency, not simply its occurrence. Although institutions provided us with the absolute number of male and female registrants, we focused on the percentage of registrants that were female, rather than assessing what factors explained absolute variation in male or female registration across institutions. It was inappropriate to make direct registration comparisons across institutions because they varied widely in their menu of sports offered, duration of playing season, participation fees, and other factors.

Some institutions provided only total male and female registrations but, in most cases, they provided information for each sport, sometimes more than 30 in total. Whenever information was available for each sport, we retained it so that we could assess the popularity of particular sports (see Results above). In addition, considering specific sports allowed us to remove some that did not meet the anthropological definition of sport provided at the outset of the paper (i.e., we excluded games that did not involve physical skill). Among those excluded were poker, “NCAA tournament bracket challenge”, “chili cookoff”, “rock paper scissors”, and trivia games. We also removed video games, despite that they might meet the definition of sport; including them would have very slightly increased the sex difference reported in this study. We combined different variations of the same general sport (e.g., outdoor and indoor soccer). In classifying sports as individual or team, we focused on each activity’s typical form of play so that, for example, doubles tennis was classified as tennis and thus as an individual sport. Some intramurals were described as tournaments, meaning play usually occurred during one or a few days. We generally counted tournaments in participation unless no information was provided on participants’ sex, which was the case at Texas A&M University and Northwestern University. Data were generally from the 2010–2011 academic year, although, in a few cases, they were only from Fall 2011. We obtained data on institutional enrollments from the National Center for Education Statistics [125].

Historical analyses were based only on institutions that provided at least five years of data. Furthermore, we required that the data be based on similar methods of data collection and a similar menu of intramural options. In particular, at one institution, the number of intramural registrations doubled from one year to the next, suggesting that yearly comparisons would be unwarranted. Some institutions provided yearly data summaries that were not strictly comparable to ones shown in Table 7, which were based on 2010–2011. For example, one institution apparently provided historical data on unique registrations, not total registrations.

## Supporting Information

Table S1 **Multivariate logistic regressions predicting participation in team and individual sports, American Time Use Survey 2003–2010.**
(DOCX)

Table S2 **Participation rates for sports and exercise for males and females across racial/ethnic groups and education levels, American Time Use Survey 2003–2010.**
(DOCX)
