# Supplementary material for: A Sex Difference in the Predisposition for Physical Competition: Males Play Sports Much More than Females Even in the Contemporary U.S
Source: PLoS One. 2012 Nov 14;7(11):e49168. doi: 10.1371/journal.pone.0049168 (PMC3498324; doi:10.1371/journal.pone.0049168)
Supplement: Table S1 — Multivariate logistic regressions predicting participation in team and individual sports, American Time Use Survey 2003–2010. (DOCX) [file pone.0049168.s001.docx]

**Table S1**. Multivariate logistic regressions predicting participation in team and individual sports, American Time Use Survey 2003-2010.^a^

| **Team Sports** | | | | **Individual Sports** | | |
| --- | --- | --- | --- | --- | --- | --- |
| **Variables** | **Odds Ratio** | **95% Wald CI** | | **Odds Ratio** | **95% Wald CI** | |
| Male | 4.27 | 4.27 | 4.28 | 2.72 | 2.72 | 2.72 |
| Female | 1.00 | -- | -- | 1.00 | -- | -- |
| **Education** |  |  |  |  |  |  |
| Less than high school | 1.00 | -- | -- | 1.00 | -- | -- |
| High school graduate | 0.49 | 0.49 | 0.49 | 1.40 | 1.40 | 1.40 |
| Some college/tech school | 0.58 | 0.58 | 0.58 | 2.07 | 2.07 | 2.07 |
| College graduate | 0.80 | 0.80 | 0.80 | 2.94 | 2.94 | 2.94 |
| **Race/ethnicity** |  |  |  |  |  |  |
| White | 1.00 | -- | -- | 1.00 | -- | -- |
| Black | 1.56 | 1.56 | 1.56 | 0.38 | 0.38 | 0.38 |
| Hispanic or Latino | 1.32 | 1.32 | 1.32 | 0.42 | 0.42 | 0.42 |
| Other race | 1.12 | 1.12 | 1.12 | 0.71 | 0.71 | 0.71 |
| **Marital status** |  |  |  |  |  |  |
| Married | 0.84 | 0.84 | 0.84 | 1.25 | 1.25 | 1.25 |
| Not married | 1.00 | -- | -- | 1.00 | --- | -- |
| **Survey year** |  |  |  |  |  |  |
| 2003 | 1.00 | -- | -- | 1.00 | -- | -- |
| 2004 | 1.10 | 1.10 | 1.10 | 1.05 | 1.05 | 1.05 |
| 2005 | 1.25 | 1.25 | 1.25 | 0.88 | 0.88 | 0.88 |
| 2006 | 1.30 | 1.30 | 1.30 | 0.90 | 0.89 | 0.90 |
| 2007 | 1.01 | 1.01 | 1.01 | 1.03 | 1.03 | 1.03 |
| 2008 | 1.38 | 1.38 | 1.38 | 0.91 | 0.91 | 0.91 |
| 2009 | 1.40 | 1.40 | 1.40 | 0.87 | 0.87 | 0.87 |
| 2010 | 1.35 | 1.35 | 1.35 | 0.78 | 0.78 | 0.78 |

^a^Models were adjusted for age group.
