# Supplementary material for: A Sex Difference in the Predisposition for Physical Competition: Males Play Sports Much More than Females Even in the Contemporary U.S
Source: PLoS One. 2012 Nov 14;7(11):e49168. doi: 10.1371/journal.pone.0049168 (PMC3498324; doi:10.1371/journal.pone.0049168)
Supplement: Table S2 — Participation rates for sports and exercise for males and females across racial/ethnic groups and education levels, American Time Use Survey 2003–2010. (DOCX) [file pone.0049168.s002.docx]

**Table S2**. Participation rates for sports and exercise for males and females across racial/ethnic groups and education levels, American Time Use Survey 2003-2010.

|  |  | **Team Sports** | **Individual Sports** | **Total Sports** | **Exercise** |
| --- | --- | --- | --- | --- | --- |
|  |  | **%** | **%** | **%** | **%** |
| **Race/ethnicity** |  |  |  |  |  |
|  | Male | 1.95 | 2.18 | 4.08 | 14.06 |
| non-Hispanic White | Female | 0.61 | 0.82 | 1.42 | 14.79 |
|  | % Females | 25.13 | 28.59 | 27.1 | 52.87 |
|  | Male | 4.85 | 0.75 | 5.60 | 12.97 |
| non-Hispanic Black | Female | 0.48 | 0.27 | 0.74 | 8.36 |
|  | % Females | 10.69 | 30.88 | 13.84 | 44.03 |
|  | Male | 2.74 | 2.39 | 5.13 | 17.54 |
| Asian/Native Hawaiian | Female | NA | NA | 1.01 | 17.39 |
|  | % Females | 15.57 | 20.78 | 18.08 | 52.63 |
|  | Male | 4.51 | 0.79 | 5.27 | 13.12 |
| Hispanic | Female | 0.90 | NA | 1.14 | 10.53 |
|  | % Females | 15.95 | 22.31 | 16.96 | 43.14 |
| **Educational attainment** |  |  |  |  |  |
|  | Male | 8.47 | 1.40 | 9.78 | 12.62 |
| <high school | Female | 2.21 | 0.57 | 2.74 | 10.06 |
|  | % Females | 20.38 | 28.72 | 21.57 | 43.96 |
|  | Male | 1.23 | 1.29 | 2.47 | 9.67 |
| high school | Female | 0.23 | 0.49 | 0.71 | 10.44 |
|  | % Females | 16.53 | 28.69 | 23.35 | 53.29 |
|  | Male | 1.67 | 1.95 | 3.59 | 12.41 |
| some college | Female | 0.25 | 0.69 | 0.93 | 12.49 |
|  | % Females | 14.88 | 29.42 | 23.55 | 54.33 |
|  | Male | 0.95 | 2.58 | 3.51 | 21.09 |
| college graduate | Female | 0.40 | 0.92 | 1.31 | 20.27 |
|  | % Females | 30.46 | 26.94 | 27.95 | 49.93 |

NA = values %/SE > 0.30.
